# The overall and sex- and age-group specific incidence rates of cancer in people with schizophrenia: a population-based cohort study

**DOI:** 10.1017/S204579602000044X

**Published:** 2020-05-28

**Authors:** D. Pettersson, M. Gissler, J. Hällgren, U. Ösby, J. Westman, W. V. Bobo

**Affiliations:** 1 Department of Neurobiology, Care Sciences and Society, Karolinska Institutet, Stockholm, Sweden; 2 Finnish Institute for Health and Welfare (THL), Helsinki, Finland; 3 Academic Primary Health Care Centre, Region Stockholm, Sweden; 4 Department of Psychiatry & Psychology, Mayo Clinic, Jacksonville, Florida, USA

**Keywords:** Cancer, comorbidity, epidemiology, schizophrenia

## Abstract

**Aims:**

Decades of research show that people with schizophrenia have an increased risk of death from cancer; however, the relationship between schizophrenia and cancer incidence remains less clear. This population-based study investigates the incidence of seven common types of cancer among people with a hospital diagnosis of schizophrenia and accounting for the effects of age, sex and calendar time.

**Methods:**

This population-based study used 1990–2013 data from three nationwide Swedish registries to calculate the incidence (in total, by age group and by sex) of any cancer and of lung, oesophageal, pancreatic, stomach, colon, (in men) prostate and (in women) breast cancer in 111 306 people with a hospital diagnosis of schizophrenia. The incidence in people with diagnosed schizophrenia was compared with the incidence in the general population. Risk estimates accounted for the effects of calendar time.

**Results:**

In 1 424 829 person-years of follow-up, schizophrenia did not confer an overall higher cancer risk (IRR 1.02, 95% CI 0.91–1.13) but was associated with a higher risk for female breast (IRR 1.19, 95% CI 1.12–1.26), lung (IRR 1.42, 95% CI 1.28–1.58), oesophageal (IRR 1.25, 95% CI 1.07–1.46) and pancreatic (IRR 1.10, 95% CI 1.01–1.21) and a lower risk of prostate (IRR 0.66, 95% CI 0.55–0.79) cancer. Some age- and sex-specific differences in risk were observed.

**Conclusions:**

People with schizophrenia do not have a higher overall incidence of cancer than people in the general population. However, there are significant differences in the risk of specific cancer types overall and by sex calling for efforts to develop disease-specific prevention programmes. In people with schizophrenia, higher risk generally occurs in those <75 years.

## Introduction

Schizophrenia is a severe and persistent mental health disorder that affects more than 23 million people worldwide (WHO 2018) and is associated with cognitive dysfunction and poor functional capacity in nearly all domains (Harvey and Strassing, 2012). People with schizophrenia also have a 20% shorter life span than people in the general population (Newman and Bland, 1991), and the mortality gap between people with schizophrenia and the general population is increasing (Saha *et al*., 2007). Although rates of completed suicide are higher in people with schizophrenia than in the general population (Baxter and Appleby, 1999), the greatest contributor to the heightened risk of premature death in people with schizophrenia is early mortality due to chronic diseases (Ösby *et al*., 2000).

The largest contributors to chronic disease-related deaths in people with schizophrenia are cardiovascular diseases and cancer (Crump *et al*., 2013; Polednak, 2014; Westman *et al*., 2018). Observational research conducted over the past three decades has consistently linked schizophrenia with an increased risk of cancer mortality (Zhuo *et al*., 2017; Tanskanen *et al*., 2018). Studies have shown that the occurrence of risk factors for cancer, such as heavy smoking, drug and alcohol abuse, poor diet and lack of exercise, in people with schizophrenia is higher than in the general population (Brown *et al*., 1999; Hartz *et al*., 2014; Hunt *et al*., 2018; Jakobsen *et al*., 2018). People with schizophrenia may also have compromised access to general health care resources (Brown *et al*., 1999; Mitchell *et al*., 2012).

However, research results on the relationship between schizophrenia and cancer incidence have been less consistent than results on the relationship between schizophrenia and cancer mortality (Hodgson *et al*., 2010). A number of early studies reported unexpectedly low rates of cancer in people with schizophrenia despite an over-representation of cancer risk factors (Mortensen, 1989, 1994; Gulbinat *et al*., 1992; Cohen *et al*., 2002), which gave rise to the hypothesis that schizophrenia or the medications used to treat it may protect against cancer risk (Mortensen, 1989; Gulbinat *et al*., 1992; Fond *et al*., 2012). A recent meta-analysis of 16 observational studies – with a total of 480 356 participants and 14 999 cases of cancer – reported a statistically significant but small decrease in the overall risk of cancer in patients with schizophrenia (RR 0.90, 95% CI 0.81–0.99) (Li *et al*., 2018). However, the results of individual studies are inconsistent. Some report lower (Barak *et al*., 2005; Dalton *et al*., 2005; Grinshpoon *et al*., 2005; Chou *et al*., 2011; Ji *et al*., 2012; Whitley *et al*., 2012; Kisely *et al*., 2016) while others report similar (Saku *et al*., 1995; Oksbjerg Dalton *et al*., 2003; Goldacre *et al*., 2005; Truyers *et al*., 2011; Lin *et al*., 2013a; Osborn *et al*., 2013; Raviv *et al*., 2014; Brink *et al*., 2019) or higher (Ananth and Burnstein, 1977; Lawrence *et al*., 2000; Lichtermann *et al*., 2001) overall cancer risks in people with diagnosed schizophrenia than in control populations.

The discrepant findings from studies of schizophrenia and cancer incidence may be explained by inter-study heterogeneity and failure to account for the numerous factors that contribute to it (Li *et al*., 2018). In particular, investigations of schizophrenia and the risk of multiple cancer types combined as a single endpoint have been criticised because individual types of cancer have heterogeneous risk factors, aetiologies and prognoses (Hodgson *et al*., 2010). As such, the risk of specific cancer types in people with schizophrenia has been the focus of several more recent epidemiological studies. In general, meta-analyses of studies that have focused on individual types of cancer suggest that the risk of breast cancer may be increased in people with schizophrenia (Catala-Lopez *et al*., 2014; Chou *et al*., 2016; Xiping *et al*., 2018; Zhuo and Triplett, 2018), although results are inconsistent (Li *et al*., 2018), and data on the associations between schizophrenia and other cancer types are equivocal or more sparse (Busche and Hodgson, 2010; Xu *et al*., 2017; Li *et al*., 2018; Zhuo *et al*., 2019). Therefore, additional studies of the effects of schizophrenia on site-specific cancer risk are needed to achieve a more comprehensive understanding of the incidence of lifestyle-related cancers in people with schizophrenia, and to confirm or identify new associations with specific cancer subtypes. This information is important for informing efforts to develop effective disease-specific preventive interventions and programmes (Chou *et al*., 2016).

We conducted a population-based study of the incidence of seven common types of cancer in 111 306 people with a hospital diagnosis of schizophrenia, using three comprehensive registries in Sweden and accounting for the effects of age, sex and calendar time. We chose to study a number of specific cancer sites either characterised by high mortality in the Swedish population or an association with adverse lifestyle factors that are also over-expressed in people with schizophrenia (e.g. smoking, alcohol consumption, physical inactivity and poor diet), or both (Regier *et al*., 1990; Hung *et al*., 2014; Maremmani *et al*., 2017; Vancamfort *et al*., 2017; Firth *et al*., 2018; Jakobsen *et al*., 2018). We hypothesised that cancers associated with these specific lifestyle factors are more common in people with schizophrenia than in the general population.

## Methods

### Study population and data sources

This study compared the incidence of cancer in people in Sweden diagnosed with schizophrenia with the incidence of cancer in the total population of Sweden between 1990 and 2013. Schizophrenia diagnoses were obtained from the National Patient Register and incident cancer diagnoses from three nationwide registers: the Cancer Register, the National Patient Register and the Cause of Death Register.

### Identification of patients with schizophrenia

The National Patient Register, which contains comprehensive information on psychiatric inpatient care nationwide from 1973 onward, was used to identify all people 15 years or older who were admitted to the hospital and received a main diagnosis of schizophrenia between 1 January 1973 and 31 December 2013 (*n* = 137 535). Diagnoses of schizophrenia were identified using WHO's International Statistical Classification of Diseases (ICD) ICD-8 and ICD-9 diagnosis codes 295, 297 and 298 (1973–1996) and ICD-10 diagnosis codes F20 to F29 (1997–2013). When measured as the proportion of true cases, using chart review as the gold standard, coverage of schizophrenia diagnoses in the National Patient Register is 84% (Dalman *et al*., 2002; Nesvåg *et al*., 2017).

### Identification of incident cancer cases

Information on cancer diagnoses from 1 January 1973 through 31 December 2013 was obtained from the Swedish Cancer Register, National Patient Register and Cause of Death Register (Ludvigsson *et al*., 2016). Individual-level data from these sources were linked via a pseudonymised version of the personal identification number, assigned to all Swedish citizens and permanent residents. When an individual had a diagnosis of the same cancer in more than one register, the earliest diagnosis was used. We used 1 January 1973 to 31 December 1989 as a wash-out period to eliminate people diagnosed with cancer prior to the start of the study.

The Swedish Cancer Register gathers comprehensive data on diagnoses of cancer in the whole population of Sweden (Barlow *et al*., 2009). The National Patient Register includes nationwide coverage of inpatient diagnoses of somatic diseases from 1987. The Cause of Death Register, established in 1961, includes data on cause(s) of death for all people registered in Sweden, including information from death certificates about date of death and main (underlying) and additional causes of death. The ICD-7 (Cancer Register) and ICD-9 and ICD-10 codes (National Patient Register and Cause of Death Register) are given in Table 2.

### Study population and follow-up

People with diagnosed schizophrenia were followed up starting on the date of their first hospital admission for schizophrenia or 1 January 1990, whichever came last. Follow-up ended on the earliest of the following four dates: date of cancer diagnosis, date of death, date of emigration or 31 December 2013. Of the 137 535 people diagnosed with schizophrenia between 1973 and 2013, a total of 20 979 died and 5250 were diagnosed with cancer before the start of the study period in 1990, leaving 111 306 people in the schizophrenia cohort.

For the total population of Sweden (the comparison group), we obtained the number of people for each year in the study period, stratified by sex and age group, from Statistics Sweden, the Swedish national statistical agency.

### Statistical analysis

The overall and site-specific cancer incidence rates in people with schizophrenia were compared with incidence rates in the general population, each calculated as the ratio of the number of incident cases to the number of person-years of follow-up. Poisson regression models were used to compare overall and site-specific cancer incidence rates in people with schizophrenia to those of the general population by estimating incidence rate ratios (IRRs) with 95% confidence intervals (CIs). The logarithm of the person-years of follow-up was used as the offset parameter. All models were adjusted for sex, calendar year and age at the time of follow-up in 5-year groups. IRRs were also estimated separately for men and women, by study period (1990–1996, 1997–2004 and 2005–2013) and by age group (15–39, 40–65 and 65 and older).

## Results

The 111 306 people with schizophrenia contributed a total of 1 424 829 person-years of follow-up (Table 1). They were predominantly middle-aged (mean [s.d.] age, 49 ± 20 years) and female (53%).

**Table 1.** Demographic and follow-up characteristics of people with schizophrenia in Sweden, in total and by sex, from 1990 through 2013

Characteristic	Total	Men	Women
*N*, total	111 306	52 044	59 262
Age, years			
Mean (s.d.)	49.9 (19.8)	44.6 (18.3)	53.0 (20.3)
Median	45.0	41.0	51.0
Follow-up, years			
Mean (s.d.)	12.8 (8.5)	13.0 (8.5)	12.6 (8.4)
Median	12.3	12.8	11.9
Follow-up, total person-years	1 424 829	678 656	746 829

s.d., standard deviation.

### Overall incidence of cancer

Among those with diagnosed schizophrenia, a total of 11 670 cases of cancer were identified (Table 2). There was no significant difference in the overall incidence of cancer compared to the general population (IRR 1.02, 95% CI 0.91–1.13). No significant differences in IRRs of overall cancer were found by age group or period (data not shown).

**Table 2.** Incidence rate ratios (IRRs) of cancer in people with schizophrenia in Sweden from 1990 through 2013^a^

Cancer site	Cancer cases (*n*)	IRR (95% CI)
Any site		
Men	4573	0.93 (0.89–0.98)
Women	7097	1.11 (1.08–1.15)
Total	11 670	1.02 (0.91–1.13)
Lung		
Men	722	1.49 (1.35–1.63)
Women	769	1.40 (1.27–1.55)
Total	1491	1.42 (1.28–1.58)
Oesophagus		
Men	106	1.29 (1.05–1.58)
Women	54	1.16 (0.91–1.47)
Total	160	1.25 (1.07–1.46)
Pancreas		
Men	207	1.22 (1.07–1.39)
Women	285	1.03 (0.92–1.16)
Total	492	1.10 (1.01–1.21)
Stomach		
Men	178	1.13 (0.97–1.31)
Women	146	0.95 (0.80–1.12)
Total	324	1.03 (0.92–1.17)
Colon		
Men	366	0.91 (0.81–1.01)
Women	610	0.97 (0.89–1.05)
Total	976	0.94 (0.87–1.01)
Prostate		
Men	1111	0.66 (0.55–0.79)
Breast		
Women	2189	1.19 (1.12–1.26)

a IRRs were estimated by using Poisson regression models comparing overall and site-specific cancer incidence rates in people with schizophrenia to those of the general population. The reference group was the general population of Sweden. Lung: ICD-7: 1621, ICD-9: 162C-162D, 162W, 162X, ICD-10: C34; Esophagus: ICD-7: 150, ICD-9: 150, ICD-10: C15; Pancreas: ICD-7: 157, ICD-9: 157, ICD-10: C25; Stomach: ICD-7: 151, ICD-9: 151, ICD-10: C16; Colon: ICD-7: 153, ICD-9: 153, ICD-10: C18; Prostate: ICD-7: 177, ICD-9: 185, ICD-10: C61; Breast: ICD-7: 170, ICD-9: 174, ICD-10: C50.

Sex-stratified analyses showed a slightly decreased incidence in men with schizophrenia (IRR 0.93, 95% CI 0.89–0.98) and a slightly increased incidence in women with schizophrenia (IRR 1.11, 95% CI 1.08–1.15). The incidence of cancer was lower in men with schizophrenia than in men in the general population during the third period (2005–2013, IRR 0.89, 95% CI 0.81–0.96) but not the first two (1990–1996 and 1997–2004). The incidence of cancer remained higher in women with schizophrenia than in women in the general population during all three periods (data not shown).

**Fig. 1.**
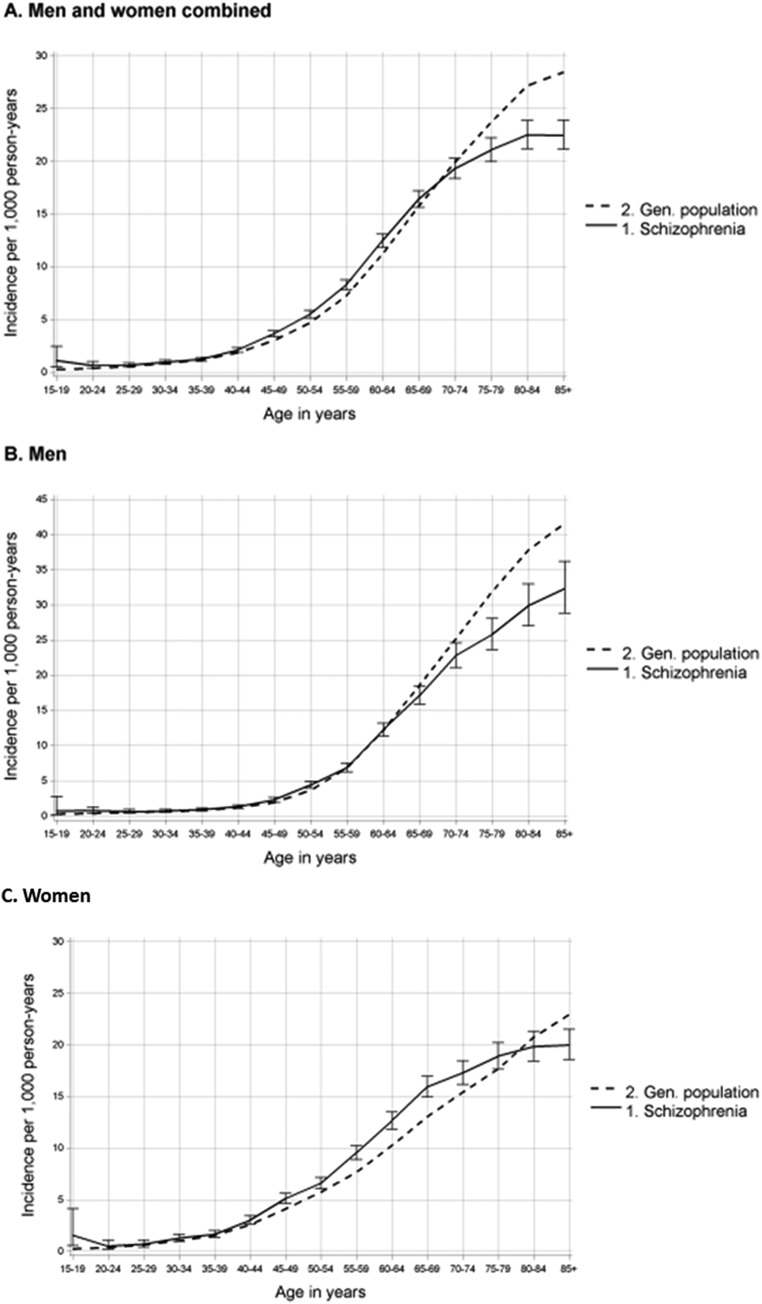
Incidence rate ratios (IRRs) of any cancer in people with schizophrenia and in the general population by age and sex,1990 through 2013.

The overall incidence of cancer in the age group 75 years and older was lower in people with schizophrenia (IRR 0.90, 95% CI 0.86–0.94). This pattern can also be seen in Fig. 1*a*. When stratified by sex, the overall incidence of cancer was lower in men with schizophrenia in the age group 65 years and older (IRR 0.86, 95% CI 0.80–0.92) (Fig. 1*b*). However, after an additional analysis that excluded prostate cancer, the lower incidence in this age group disappeared (IRR 0.96, 95% CI 0.92–1.02), and in the 40–64 years age group, a higher cancer incidence was observed among men with schizophrenia (IRR 1.26, 95% CI 1.19–1.33). The overall incidence of cancer was significantly higher in women with schizophrenia who were 40–64 years (IRR 1.22, 95% CI 1.16–1.28) and 65 years and older (IRR 1.05, 95% CI 1.02–1.09), than in the general population (Fig. 1c).

### Site-specific incidence of cancer

People with schizophrenia had a significantly higher incidence of cancers of the lung, oesophagus, pancreas and breast than people in the general population (Table 2). On the other hand, men with schizophrenia had a lower incidence of prostate cancer (IRR 0.66, 95% CI 0.55–0.79). There was no significant association between schizophrenia and incidence of cancer of the stomach or colon. Significantly higher incidence of oesophageal cancer and of pancreatic cancer was observed in men only.

After stratifying for time period (Table 3), we found that women with schizophrenia had an excess risk of breast cancer in all three periods (1990–1996, IRR 1.22, 95% CI 1.09–1.37; 1997–2004, IRR 1.17, 95% CI 1.06–1.30; and 2005–2013, IRR 1.18, 95% CI 1.08–1.30). The incidence of lung cancer was elevated in people with schizophrenia during the second (IRR 1.37, 95% CI 1.14–1.65) and third (IRR 1.64, 95% CI 1.41–1.89) periods of the study. Similarly, the incidence of oesophageal cancer was significantly higher in people with schizophrenia during the second (IRR 1.39, 95% CI 1.08–1.79) and third (IRR 1.29, 95% CI 1.01–1.63) study periods. The incidence of prostate cancer was decreased in men with schizophrenia during the second (IRR 0.67, 95% CI 0.48–0.93) and third (IRR 0.60, 95% CI 0.45–0.80) study periods.

**Table 3.** Incidence rate ratios (IRRs) of cancer in people with schizophrenia in Sweden from 1990 through 2013 by period^a^

	1990–1996				1997–2004				2005–2013			
	Cancer cases	IRR	95% CI		Cancer cases	IRR	95% CI		Cancer cases	IRR	95 %	
Any site	3202	1.07	0.88	1.31	3752	1.00	0.83	1.20	4716	0.99	0.84	1.17
Lung	298	1.12	0.89	1.41	458	1.37	1.14	1.65	735	1.64	1.41	1.89
Oesophagus	33	1.00	0.71	1.40	59	1.39	1.08	1.79	68	1.29	1.01	1.63
Pancreas	133	1.07	0.90	1.27	158	1.11	0.95	1.30	201	1.12	0.98	1.29
Stomach	108	0.97	0.78	1.20	101	0.97	0.78	1.21	115	1.18	0.96	1.45
Colon	263	0.95	0.83	1.09	292	0.87	0.76	0.99	421	0.99	0.89	1.10
Prostate(men)	262	0.80	0.54	1.19	366	0.67	0.48	0.93	483	0.60	0.45	0.80
Breast(women)	573	1.22	1.09	1.37	728	1.17	1.06	1.30	888	1.18	1.08	1.30

a IRRs were estimated by using Poisson regression models comparing overall and site-specific cancer incidence rates in people with schizophrenia to those of the general population. The reference group was the general population of Sweden.

In analyses that were stratified by age group, the incidence of lung cancer was elevated in people with schizophrenia across all groups, but the incidence estimates decreased with increasing age strata (15–39 years, IRR 2.49, 95% CI 1.50–4.11; 40–64 years, IRR 1.90, 95% CI 1.67–2.15; and 65 years or older, IRR 1.21, 95% CI 1.05–1.39). There was an excess incidence of breast cancer in women with schizophrenia aged 40–64 years (IRR 1.19, 95% CI 1.09–1.29) and 65 years and older (IRR 1.21, 95% CI 1.10–1.32). The incidence of pancreatic cancer was significantly increased in people with schizophrenia between the ages of 15 and 39 years (IRR 2.93, 95% CI 1.16–2.31) and 40–64 years (IRR 1.31, 95% CI 1.10–1.56). The incidences of oesophageal (IRR 1.53, 95% CI 1.21–1.93) and stomach cancer (IRR 1.43, 95% CI 1.17–1.74) were significantly higher in people with schizophrenia only in the 40–64 years age group. The incidence of prostate cancer was significantly lower in men with schizophrenia who were 40–64 (IRR 0.54, 95% CI 0.44–0.66) and 65 or older (IRR 0.70, 95% CI 0.56–0.88), whereas the incidence of colon cancer was significantly lower in people with schizophrenia only in those 65 years or older (IRR 0.90, 95% CI 0.83–0.97).

## Discussion

### Main findings

In this population-based study, we found the same overall risk of cancer in people with schizophrenia as in the general population. However, we found a higher overall cancer risk among women with schizophrenia. The overall cancer risk for men was reduced among men with schizophrenia but the reduced risk disappeared when excluding prostate cancer from the analysis. We found a pattern of relative incidence rates among schizophrenia patients being higher in lower age groups when stratifying the analyses for age. A notable exception was observed for prostate cancer, where relative incidence rates were lower among younger men with schizophrenia. People with schizophrenia had a higher risk of lung, oesophageal and pancreatic cancer, women with schizophrenia had a higher risk of breast cancer, and men with schizophrenia had a lower risk of prostate cancer than the general population. Also some noteworthy differences in the overall and site-specific incidences of cancer by age category and by sex were observed.

The increased relative incidences in the schizophrenic population observed for lung, oesophageal, pancreatic and breast cancer were expected due to the higher prevalence of heavy smoking, alcohol use, obesity and lack of exercise in this population (Brown *et al*., 1999; von Hausswolff-Juhlin *et al*., 2009; Hartz *et al*., 2014; Hunt *et al*., 2018; Jakobsen *et al*., 2018). The decreased incidence of prostate cancer compared to the general population can probably be explained by lower rates of detection by opportunistic prostate cancer screening. Prostate cancer screening is not part of the national screening programme in Sweden but testing is available at low cost within the Swedish health care system at patient's request.

### Comparison with previous studies

Our finding of no excess overall risk of cancer associated with schizophrenia is broadly consistent with the findings of a meta-analysis of six studies that was published in 2008 (Catts *et al*., 2008) but contrasts with the results of a more recent and larger meta-analysis of 16 cohort studies that found a small but statistically significant decreased risk of cancer in people with schizophrenia (Li *et al*., 2018). The inconsistencies in the conclusions of these two meta-analyses reflect the inconsistencies in the results of the individual studies. Indeed, in the meta-analysis by Li *et al.* (2018), the percentage of total variation across studies was more than 90%, which indicates a high degree of inconsistency in the results of the individual studies (Higgins *et al*., 2003). Li *et al.* (2018) advised that incidence risk ratios should be calculated for individual cancer types and separately for men and women.

Similar to the pooled analyses (Li *et al*., 2018) and an Israeli study (Agay *et al*., 2017), we found a significantly lower risk of prostate cancer in men and a significantly higher risk of lung cancer in women with schizophrenia. In line with the findings from two meta-analyses of observational studies that focused on the association between schizophrenia and female breast cancer (Xiping *et al*., 2018; Zhuo and Triplett, 2018), we found a higher incidence of breast cancer in women with schizophrenia. Our findings are also consistent with two more recently-published population-based cohort studies that documented significantly higher risk of newly-diagnosed breast cancer in women with schizophrenia (Wu *et al*., 2017; Chen *et al*., 2018). Few additional noteworthy sex differences in the risk of specific cancers in people with schizophrenia were observed in our study. In summary, the observations in our study are broadly consistent with recently-published pooled analyses of data from cohort studies investigating the sex-specific risks of individual cancer subtypes in people with schizophrenia.

Some of the cancer types that were the focus of this study were chosen based primarily on their association with adverse lifestyle factors that are also highly-prevalent in people with schizophrenia. For instance, a population-based retrospective cohort study of people with diagnosed mental health disorders found significant associations between female breast cancer and alcohol use and the presence of metabolic syndrome; between lung cancer and tobacco use; between oesophageal cancer and tobacco and alcohol use; and between pancreatic cancer and the use of tobacco (Hung *et al*., 2014). These results are consistent with the extensive literature that identifies lifestyle factors such as unhealthy diet, excessive body weight, physical inactivity, excessive alcohol consumption and smoking behaviours as modifiable risk factors for developing breast, prostate, lung and colon cancers in the broader population (Khan *et al*., 2010; Weiderpass, 2010) and for reducing cancer-related deaths (Islami *et al*., 2018). We lacked specific information on the prevalence of unhealthy lifestyle-related risk factors among people with schizophrenia residing in Sweden. A national register study that used 1987–2010 data from the entire Swedish population aged 15 years or older showed that people with diagnosed schizophrenia had a threefold higher mortality from cardiovascular diseases than people in the general population (Westman *et al*., 2018). Inasmuch as smoking, alcohol consumption, poor diet and sedentary lifestyle are risk factors common to both cardiovascular diseases and lifestyle-related cancers, the high prevalence of these risk factors in people with schizophrenia in other parts of the world highlights the considerable potential for reducing the risk of lifestyle-related cancers as well as premature death from cardiovascular causes worldwide (Regier *et al*., 1990; Hung *et al*., 2014; Maremmani *et al*., 2017; Vancamfort *et al*., 2017; Firth *et al*., 2018; Jakobsen *et al*., 2018).

Our study found that people with schizophrenia had a significantly higher incidence of oesophageal cancer. The risk was significantly increased for men but not women. In a nested case–control study that included 40 441 incident cases of six different types of cancer, researchers found no higher risk of gastroesophageal cancer in people with schizophrenia (Hippisley-Cox *et al.*, 2007). In that study, gastric and oesophageal cancers were combined, whereas in our study, they were analysed separately. No other population-based studies have reported the comparative risks of oesophageal cancer, discretely, in people with schizophrenia.

Our study also provides new information about schizophrenia and the risk of pancreatic cancer. We observed an increased risk of pancreatic cancer in people with schizophrenia. Stratification by sex showed an increased risk in men but not in women. We are aware of only one nationwide, population-based cohort study of the risk of pancreatic cancer in people with schizophrenia (Lin *et al*., 2013*a*). That study showed a lower risk of pancreatic cancer in patients with schizophrenia than in the general population of Taiwan. They excluded metastatic malignancies from their analyses to reduce misclassification of the primary tumour site. However, because people with schizophrenia are typically diagnosed at a later stage of cancer (Kisely *et al*., 2008), excluding metastatic malignancies may have led to an underestimation of cancer diagnoses in people with schizophrenia.

In our study, we observed a decreased overall cancer risk in people with schizophrenia 75 years and older. This observation is in line with findings from prior research showing that the incidence rates of cancer in people with schizophrenia may decline with increasing age (Dalton *et al*., 2004; Lin *et al*., 2013*a*, 2013*b*). The underlying causes of this age-related trend are unknown, but may be caused by selection where the small fraction of the schizophrenia population that reaches high age are physically healthier or have less risk factors than the general population of the same age. Cancer and cardiovascular disease, the other leading cause of death in Sweden, share several risk factors, such as smoking, alcohol use, obesity and physical inactivity. If schizophrenia modifies the effect of risk factors common between cancer and cardiovascular disease such that the effect on the risk of death is larger in people with schizophrenia, the prevalence of risk factors will decrease faster among people with schizophrenia as the populations age. We suggest two possible mechanisms through which schizophrenia could modify the effect of risk factors: (1) by increasing the probability of a dichotomous distribution of the risk factor, for example, smokers tending to be heavy smokers and obese people tending to be severely obese; and (2) increasing the probability of having a combination of risk factors. However, the Swedish national health register data do not include information on exposure to such lifestyle factors and we did not address the relative impact of early mortality from non-cancer-related causes on the incidence of new cancer diagnoses in this work.

Several lines of evidence suggest that people with schizophrenia may experience accelerated ageing (Kirkpatrick *et al*., 2008; Kirkpatrick and Kennedy, 2018). These include earlier manifestations of cognitive decline, diabetes and other age-associated chronic diseases (Kirkpatrick *et al*., 2008), as well as the early expression of a wide variety of biomarkers related to the ageing process (Kirkpatrick and Kennedy, 2018). It is not clear whether a potentially heightened risk of some types of cancers in younger and middle-aged people with schizophrenia can be attributed to accelerated ageing.

### Strengths and limitations

The Swedish Cancer Register has nationwide coverage (Barlow *et al*., 2009) and is well-suited for measuring cancer incidence in the general population. However, the register's completeness of incidence in people with schizophrenia may be limited. Underreporting is more likely for those whose histology or cytology verification is missing, which most commonly occurs when cancer is diagnosed at an advanced stage and the patient receives only palliative care (Barlow *et al*., 2009). People with schizophrenia have low rates of participation in general health and cancer screening programmes (Fujiwara *et al*., 2017; Hwong *et al*., 2020; Solmi *et al*., 2020), and delayed and late-stage cancer diagnoses are common in this patient population (Farasatpour *et al*., 2013). To compensate the underreporting in the Swedish Cancer Register and minimise possible differential completeness in reporting, we used additional data on incident cancers from other nationwide registers, including the National Patient Register or the Swedish Cause of Death Register.

To the best of our knowledge, the current study is the largest to date with respect to the number of individuals with diagnosed schizophrenia. An additional strength of the study was the analysis by calendar period of follow-up. The mean (49 years) and median (45 years) age and age span (up to more than 80 years) of the schizophrenia cohort in the study were also advantageous, as they allowed us to compare incidence in age groups for which the risk of incident cancer is most relevant, including advanced age.

There are also limitations to consider. This study used data from computerised, linked registers. These data were collected for purposes other than for research, and may be subject to misclassification. Additionally, given that this was a register-based study, our findings are restricted to the incidence of diagnosed cancer. Even though the underlying population in this study had universal health coverage, the true incidence of cancer is likely obscured by low participation in general preventive health services and cancer screening programmes that is frequently observed in people with schizophrenia (Mitchell *et al*., 2012; Liu *et al*., 2017; Irwin *et al*., 2019). This bias would be especially problematic when studying cancers with less acute courses (e.g. prostate, skin or thyroid cancer), while this bias would be less of a concern when studying cancers that are often detected when acute symptoms are already present (e.g. lung, oesophagus and stomach cancers). Although the schizophrenia cohort in this study was large, because of sample size limitations, we were not able to study the comparative risks of all cancer types, including important but rarer malignancies. Some of the statistically significant associations between schizophrenia and some site-specific cancers, such as the results on oesophageal and pancreatic cancer, must be interpreted with caution given small effects (IRR near 1) and evidence of low precision (wide 95% CIs). We were unable to examine the impacts of a variety of susceptibility factors on cancer incidence in this study, including family history, genetic characteristics, use of antipsychotic drugs and other medications, reproductive history and lifestyle factors. We suggest national health information systems should include comprehensive registration on primary health care visits, and collection of these data should include also information on BMI, smoking and alcohol consumption. Further, our data lacked information on the stage of cancer. This is an important consideration given the likelihood that people with schizophrenia present with a more advanced stage of cancer at the time of diagnosis (Toender *et al*., 2018) – a factor that has been associated with worse cancer survival in people with pre-existing mental disorders (Davis *et al*., 2020). Others have documented a significantly higher proportion of individuals with evidence of metastatic disease at presentation in people with psychiatric illness (Kisely *et al*., 2013). The availability of information on cancer stage at diagnosis will be an important consideration for future population-based studies of cancer incidence and survival in people with severe mental illnesses as it could add information about the mechanisms behind the high cancer mortality among schizophrenia patients. In our study, follow-up was available until 2013, and we did not have complete data for the analysis of cancer risk beyond that time. Finally, all study data came from the population of Sweden, so the risk estimates may not generalise to people living in other parts of the world.

## Conclusions

There was no overall higher risk of cancer in people with schizophrenia than in the general population in this population-based study. However, people with schizophrenia had a higher risk of lung, oesophageal and pancreatic cancer; women with schizophrenia had a higher risk of breast cancer; and men with schizophrenia had a lower risk of prostate cancer than the equivalent groups of people in the general population. The observed differences in incidence between schizophrenia patients and the general population correspond to known differences in lifestyle factors or, for prostate cancer, expected differences in tumour detection through differential participation in opportunistic screening outside of the national screening programmes. Some noteworthy sex-specific differences in cancer risk were observed. A lower overall risk of cancer was observed in people with schizophrenia aged 75 years or more than in the same age group in the general population.

## Data

The Swedish register data have been given for this specific study, and the data cannot be shared without authorisation from the register keepers.
